# A Chatbot for Perinatal Women’s and Partners’ Obstetric and Mental Health Care: Development and Usability Evaluation Study

**DOI:** 10.2196/18607

**Published:** 2021-03-03

**Authors:** Kyungmi Chung, Hee Young Cho, Jin Young Park

**Affiliations:** 1 Department of Psychiatry, Yonsei University College of Medicine Yongin Severance Hospital Yonsei University Health System Yongin-si Republic of Korea; 2 Center for Digital Health Yongin Severance Hospital Yonsei University Health System Yongin-si Republic of Korea; 3 Institute of Behavioral Science in Medicine Yonsei University College of Medicine Yonsei University Health System Seoul Republic of Korea; 4 Department of Obstetrics and Gynecology CHA Gangnam Medical Center CHA University Seoul Republic of Korea

**Keywords:** chatbot, mobile phone, instant messaging, mobile health, perinatal care, usability, user experience, usability testing

## Abstract

**Background:**

To motivate people to adopt medical chatbots, the establishment of a specialized medical knowledge database that fits their personal interests is of great importance in developing a chatbot for perinatal care, particularly with the help of health professionals.

**Objective:**

The objectives of this study are to develop and evaluate a user-friendly question-and-answer (Q&A) knowledge database–based chatbot (Dr. Joy) for perinatal women’s and their partners’ obstetric and mental health care by applying a text-mining technique and implementing contextual usability testing (UT), respectively, thus determining whether this medical chatbot built on mobile instant messenger (KakaoTalk) can provide its male and female users with good user experience.

**Methods:**

Two men aged 38 and 40 years and 13 women aged 27 to 43 years in pregnancy preparation or different pregnancy stages were enrolled. All participants completed the 7-day-long UT, during which they were given the daily tasks of asking Dr. Joy at least 3 questions at any time and place and then giving the chatbot either positive or negative feedback with emoji, using at least one feature of the chatbot, and finally, sending a facilitator all screenshots for the history of the day’s use via KakaoTalk before midnight. One day after the UT completion, all participants were asked to fill out a questionnaire on the evaluation of usability, perceived benefits and risks, intention to seek and share health information on the chatbot, and strengths and weaknesses of its use, as well as demographic characteristics.

**Results:**

Despite the relatively higher score of ease of learning (EOL), the results of the Spearman correlation indicated that EOL was not significantly associated with usefulness (ρ=0.26; *P*=.36), ease of use (ρ=0.19; *P*=.51), satisfaction (ρ=0.21; *P*=.46), or total usability scores (ρ=0.32; *P*=.24). Unlike EOL, all 3 subfactors and the total usability had significant positive associations with each other (all ρ>0.80; *P*<.001). Furthermore, perceived risks exhibited no significant negative associations with perceived benefits (ρ=−0.29; *P*=.30) or intention to seek (SEE; ρ=−0.28; *P*=.32) or share (SHA; ρ=−0.24; *P*=.40) health information on the chatbot via KakaoTalk, whereas perceived benefits exhibited significant positive associations with both SEE and SHA. Perceived benefits were more strongly associated with SEE (ρ=0.94; *P*<.001) than with SHA (ρ=0.70; *P*=.004).

**Conclusions:**

This study provides the potential for the uptake of this newly developed Q&A knowledge database–based KakaoTalk chatbot for obstetric and mental health care. As Dr. Joy had quality contents with both utilitarian and hedonic value, its male and female users could be encouraged to use medical chatbots in a convenient, easy-to-use, and enjoyable manner. To boost their continued usage intention for Dr. Joy, its Q&A sets need to be periodically updated to satisfy user intent by monitoring both male and female user utterances.

## Introduction

### Background

With a growing interest in chatbots based on various digital platforms such as websites, social channels, and mobile apps, a wide range of gratifications have been suggested as motivators of chatbot use. In general, productivity is considered to be a key factor in driving chatbot use, which means that the ease, speed, and convenience of using chatbots can help their users, who seek instant gratification via quick and consistent feedback and dialogue to obtain information or assistance in a timely and efficient manner [1]. Particularly, medical chatbots as a virtual doctor or educator have been built to reduce the burden of health care costs, improve the accessibility of medical knowledge, and empower patients with their medical decision-making process [2-8]. When it comes to developing medical chatbots using artificial intelligence (AI), a number of previous studies have focused on not only accurate prediction, diagnosis, or personalized management and treatment of diseases based on their symptoms [3,4,6-9], but also conversational agent role in social and emotional support and mental health interventions [10-16]. However, the major challenge perceived by more than 70% of the medical physicians in one study is the inability of health care chatbots to address the full extent of a patient’s needs and understand or display the emotional state of humans [17]. Furthermore, common concerns on inaccurate and inflexible information that chatbots provided have been raised [3,5,17-20]. Despite these continuous attempts to provide patients with better user experience (UX) on informational and emotional support, both costs and benefits are still associated with the use of medical chatbots.

In addition to productivity, entertainment, and social or relational benefits, there are other main motivations to use chatbots, which are considered to be more humanlike than other interactive systems designed to support enjoyable social interactions [1]. As patients with lower health literacy are more likely to use and trust informal health information sources, such as television, social media, friends, blogs, celebrity webpages, and pharmaceutical companies, than formal ones such as doctors and health professionals [21], medical chatbots are required to provide their users with evidence-based health information as answers to questions from them. Given that the majority of pregnant women tend to use multiple information sources for their antenatal and postnatal care [22,23], obtaining conflicting information can increase anxiety levels or add uncertainty on whether or not to use a medication [24]. In fact, the attention of prenatal women seeking informal information or multiple information from multiple sources can be readily directed to social and emotional support from other experienced mothers and friends who have been in a similar situation, but they can experience stigma and receive inappropriate support due to their lack of related knowledge [25]. As more and more online communities have formed with huge numbers of female members who have undergone many different situations during pregnancy and childbirth, maintaining social interaction with their peers can encourage perinatal women to satisfy their curiosity and interests in specific information and content, which is thus perceived as an immediate and enjoyable daily activity. In turn, it means that medical chatbots with the characteristics of these peers, as well as a valid, accurate, and credible medical knowledge database, can be more likely to capture perinatal women’s attention when encountering medical problems.

To encourage people to adopt and use medical chatbots, both content quality and expertise of the chatbots should be first considered in the development process. From the perspective of utilitarian and hedonic value, content quality has strongly positive effects on perceived usefulness and enjoyment, both of which influence users’ usage intention [26]. Perceived expertise of the medical chatbots can increase the users’ trust in the chatbots, which in turn affects their continuance intention to use the service agents [27]. In addition to the effort to improve a chatbot’s content quality and expertise, it is also important to iteratively evaluate its usability and UX, both in the development process and after the completion of its development. According to Lund, who developed the Usefulness, Satisfaction, and Ease of Use (USE) Questionnaire [28], ease of use and usefulness influence each other and drive satisfaction strongly related to predicted and actual usage; ease of use can be separated into two factors, ease of use (EOU) and ease of learning (EOL), if the systems to be assessed are internal systems that its users are required to use. However, it is less likely that the two factors will be highly correlated for this chatbot based on a mobile instant messenger (MIM), as it is a flexible system used in different contexts and for different needs of individuals. Furthermore, a wide range of satisfaction dimensions (ie, productivity, entertainment, social or relational benefit, etc) can serve as motivators of chatbot use [1], and therefore, there is a need to identify these motivations or any other barriers associated with the users’ intention to seek and share health information on the medical chatbot via MIM.

From the findings of a previous study based on a net valence model [29], perceived benefits were positively related to the intention to seek and share health information in social media in both Chinese and Italian samples, but only the Chinese sample showed a negative relationship between perceived risk and the intention to share health information. Until recently, little was known about the relationship between the variables in MIM-based medical chatbot use in a Korean sample. Considering that a MIM app such as KakaoTalk, which is the most popular in South Korea, is more private than other social media platforms such as YouTube, Facebook, and Twitter, it is expected that the negative relationship between the variables will not be observed in this study sample. However, it is challenging to explore the motivators and barriers to chatbot use in everyday life, not in experimental contexts, and its associations with different intention behaviors by applying a single quantitative or qualitative method, particularly in contextual usability testing (UT) without the intervention of a facilitator.

### Objectives

Taken together, the primary purpose of this study is to develop a user-centered question-and-answer (Q&A) knowledge database–based chatbot for perinatal women’s and their partners’ obstetric and mental health care by applying a text-mining technique. The secondary purpose is to evaluate it by conducting contextual UT, thereby measuring the perception of usability and UX and their associations with motivators and barriers to chatbot use and different intention behaviors and obtaining theoretical and practical implications to supplement the weaknesses of this chatbot. Based on relevant literature, we hypothesize that this chatbot will produce both utilitarian and hedonic value during the 7-day contextual UT period.

## Methods

### Chatbot Development

Dr. Joy was developed with the “kakao i” open builder, which allows businesses and users to create custom AI services provided in “KakaoTalk,” the most widely used web- and mobile-based instant messaging application in South Korea. As kakao’s AI platform could support two main features to develop a Q&A chatbot, (1) by uploading a structured Q&A Excel data file to its “knowledge+” menu or (2) by creating dialog blocks to add the users’ text input and the chatbot’s output to each scenario and linking these blocks in the “scenario” menu, both features were applied in this chatbot development. As this chatbot was only available in Korean, Multimedia Appendices 1-3 are provided to enhance Korean readers’ understanding of all figures translated to English from Korean.

#### Persona

**Figure 1:**
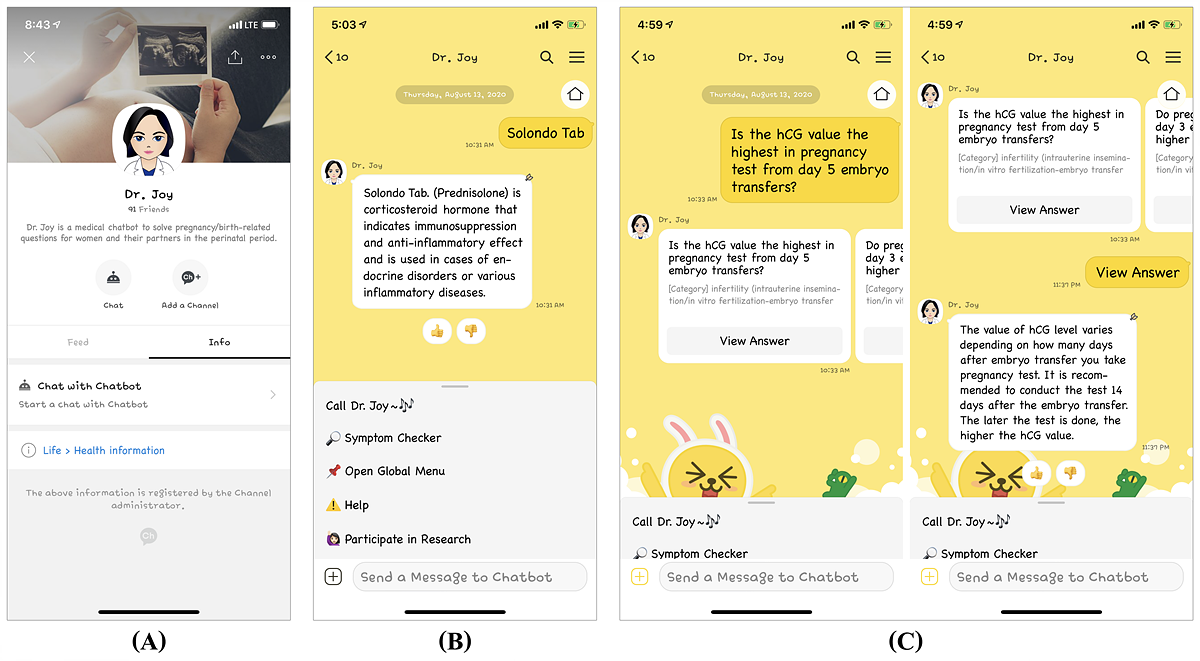
Screenshots of (A) Dr. Joy’s persona introduced on the KakaoTalk Channel and examples of (B) 1 Q&A pair and (C) 3 Q&A pairs that can be triggered when user intent matches most closely with them in Dr. Joy’s obstetric and mental health–related Q&A knowledge database.

Dr. Joy was named after the second author, whose name is pronounced similarly to “Joy,” as this chatbot was designed to lead users to perceive enjoyment when seeking health information and medical help for their prenatal and postnatal care. In order to look more professional to users, we provided Dr. Joy with a character of a “humanlike” female medical doctor (Figure 1 and Multimedia Appendix 1) and a formal, firm tone of voice, particularly when answering the questions. However, Dr. Joy demonstrated warmth in its informal, pleasant voice tone, manner, and emoji use when treating users in other scenarios.

#### Perinatal and Postnatal Care Knowledge Database

By employing the data-mining technique, the user-friendly obstetric and mental health–related Q&A knowledge database was built. A list of 3524 refined Q&A sets was created by the following procedure. To build a data set of medical questions and their terms that are of real interest and concern among Korean perinatal women, we first developed a web crawler in Python. From one of South Korea’s largest online communities for prenatal, postnatal, and maternal care, message boards with 6 different topics (ie, infertility: intrauterine insemination, in vitro fertilization, embryo transfer; pregnancy diagnosis: pregnancy test kit, ultrasound scan; pregnancy preparation; pregnancy; labor and delivery; and postpartum recovery) were chosen to be crawled, and then all posts during a 1-year period, from August 1, 2017, to August 31, 2018, were automatically collected. Contents retrieved by the web crawler were parsed into each Excel spreadsheet file by topics and stored into the following column headings: nickname (ID), timestamp (date and time), URL, title, body content (text only), and up to 3 replies.

As some contents were involved in more than one topic, all 6 topics were redefined to remove overlap. First, the topic of “pregnancy preparation” was redefined to address all posts excluding the posts on “infertility” and “pregnancy diagnosis,” and any posts irrelevant to the redefined topic were moved to the topic of either “infertility” or “pregnancy diagnosis” to effectively search questions or statements to be updated. Second, the topic of “pregnancy” was redefined to address the posts from the first to the ninth month of pregnancy because the message board about “pregnancy” covered all posts from the first to the last month of pregnancy and that about “labor and delivery” partially included posts at the tenth month of pregnancy.

From the title and body content of the posts, we extracted medical questions whose context and intent could be generally understood by both medical doctors and peer users and eliminated personal questions that were beyond a medical scope to satisfy one’s own curiosity. After that, excessively long, complex questions or statements about medical and obstetric problems were refined as simple, conversational questions or statements that one might ask a MIM-based chatbot, particularly at medium length. In the next step, to establish the data set of user-friendly question and professional answer pairs on these particular topics, a total of 11 medical doctors, who were specialized in infertility (3/11, 27%), obstetrics and gynecology (6/11, 55%), and psychiatry (2/11, 18%), were recruited; 6 (55%) and 5 (45%) of these were recruited from local hospitals and university hospitals, respectively, by using a snowball sampling method. They first identified and revised inappropriate questions or statements with false terms or without user intent and contextual information, answered all 3524 questions with a consistent tone and manner, and finally cross-checked the Q&A pairs involved in their specialty. The 3524 Q&A sets were categorized as follows: (1) infertility (intrauterine insemination, in vitro fertilization, embryo transfer: 609 items), (2) pregnancy diagnosis (pregnancy test kit, ultrasound scan, blood test: 381 items), (3) pregnancy preparation (303 items), (4) pregnancy (1-36 weeks [1-9 months]: 1154 items), (5) labor and delivery (37-40 weeks [final months]: 446 items), and (6) postpartum recovery (631 items).

Following the aforementioned procedure, we filled in the Excel spreadsheet template that the chatbot builder provided, particularly with the following data: number, category, question, and answers. In addition to the Q&A knowledge database, we built a dictionary of synonyms to improve the accuracy of providing the Q&A pairs that match well with user intent (ie, search intent), as perinatal women tend to use a wide variety of abbreviations for medical terms and neologisms in the online community. This dictionary was also organized within the given Excel template and registered into the “my entity” menu.

#### Main Features and User Interface

As a Q&A chatbot, Dr. Joy had the main feature as a bot to answer user queries and frequently asked questions. The main feature, which was developed by the Knowledge+ feature of kakao’s chatbot builder, worked by searching for questions similar to users’ dialog input in the stored Q&A knowledge database and then outputting answers linked to those questions. As shown in Figure 1 (Multimedia Appendix 1), Dr. Joy, employing an AI engine called kakao i sympson (a similarity inference engine for evaluating semantic similarity between sentences), could answer all questions by offering either (1) only 1 Q&A pair that matches the best with the user intent or (2) the 3 Q&A pairs that match most closely. Even if the given 3 Q&A pairs did not completely meet users’ intentions in asking a question to the chatbot, the users could come to know other peer mothers’ current interests and concerns from the questions and the 11 aforementioned medical doctors’ accurate, professional answers to the questions consisting of relevant medical knowledge and advice. To use this feature, users could type their questions into an input box directly or do so after calling Dr. Joy by dragging the generic menu up to open it and then tapping the button to call the chatbot. The input box and the generic menu were located at the bottom of the chatbot. Otherwise, users could also call the chatbot after accessing the graphical user interface (UI)–based global menu via the generic menu (Figure 1 and Multimedia Appendix 1).

**Figure 2:**
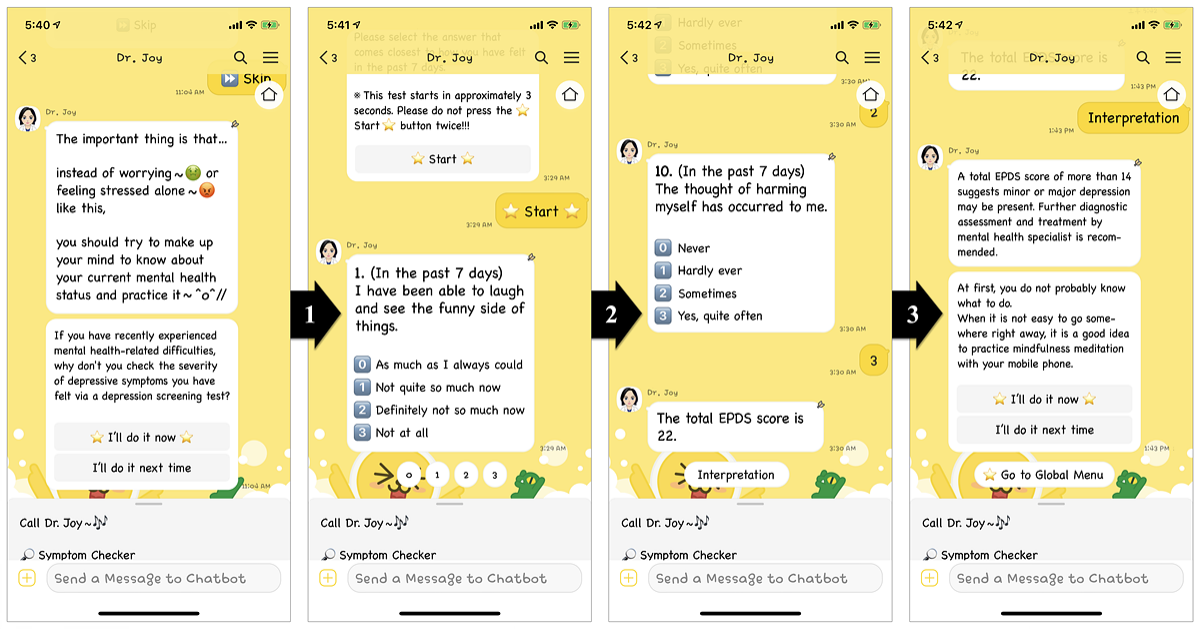
Screenshots of user interface workflow for a depression screening test using the 10-item Edinburgh Postnatal Depression Scale that can be administered in the prenatal period, followed by the screening test result and therapy suggestions.

**Figure 3:**
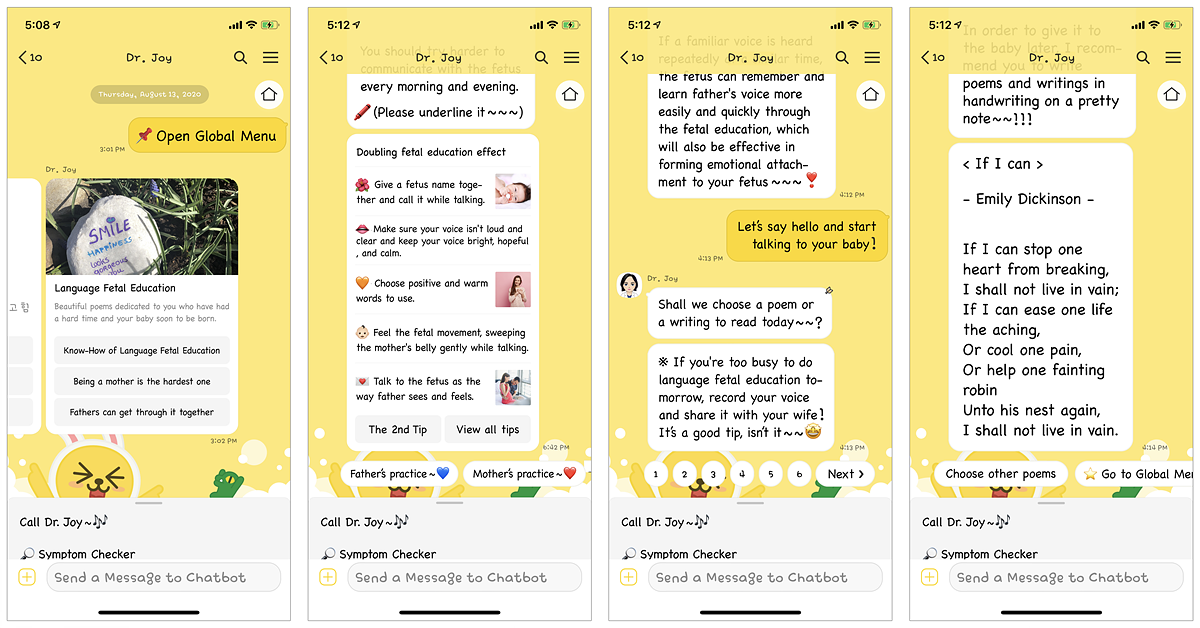
Screenshots of additional features with which male partners can provide their pregnant partners with social support that is needed for physical and mental health care, or women can take care of themselves.

With a particular focus on managing perinatal women’s mental and physical health, other main features were developed based on predefined conversational design and rule- and choice-based dialogues, which only performed and worked within scenarios. To handle unexpected responses from the users and their unwanted escape from a prearranged conversational UI flow, Dr. Joy provided the users with dialog buttons to choose as their responses to call the linked dialog blocks, particularly motivating them to follow the given UI flow. The scenario-based additional features were designed to lead the users to learn about the importance of (1) early detection of physical and obstetric problems (if users experienced specific physical symptoms, they could check up on their current health status by answering symptom-related questions that Dr. Joy asked; this chatbot-assisted medical examination was the same as a medical doctor–administered medical examination), (2) preventative mental health care, such as a depression screening test and cognitive behavioral therapy (ie, sleep hygiene education and mindfulness-based intervention; Figure 2 and Multimedia Appendix 2), and (3) social supports from their male partners, such as fetal education and various useful tips for physical and mental health care (Figure 3 and Multimedia Appendix 3).

These needs for preventative mobile health care and social supports from the perinatal women’s partners in everyday life were identified through in-depth interviews with 11 patients, 10 women and 1 man in the perinatal period, and a focus group interview with two obstetrician-gynecologist (ob/gyn) groups: (1) 3 ob/gyns at local hospitals and (2) 3 ob/gyns at university hospitals. According to the reports of the interviews, both patients and medical doctors highlighted the importance of the relationship between perinatal women and their partners on the women’s mental health during the prenatal, pregnancy, and postnatal periods. Particularly, the female interviewees and the doctors’ female patients who had experienced depressed symptoms expressed that they had a lack of opportunity to spend time with their partners in common; otherwise, a few women’s partners had cheated on them during pregnancy. By contrast, it was reported that the male interviewee, whose wife had no specific mental problems throughout pregnancy and after birth but who experienced depressed symptoms instead of her, tried to help his wife to overcome postpartum blues by sharing house chores, having a talk with her as much as possible, and ventilating her feelings of physical and emotional distress related to the double burden of childcare and housework. However, without their partners’ support, most pregnant women and mothers had difficulty in going out to refresh themselves or to attend a variety of mental health care programs held in local community health centers, local or university hospitals, and postpartum care centers. Although the male partners were also susceptible to the women’s mood fluctuations in the long-term period, both found it difficult to consult with health professionals and others (eg, family members and friends) about emotional or psychiatric problems and to consider using appropriate psychotropic medication about which a concern that it might negatively affect their fetuses might be raised. Furthermore, there has been a limitation in that the accessibility of useful information for effectively treating the women and even their partners was not significantly improved, particularly from the men’s point of view.

On the basis of these findings from the interviews, the same sample of medical doctors who had participated in the development of Dr. Joy’s Q&A knowledge contents as the main feature to answer questions regarding obstetric and mental health concerns in both perinatal women and their partners guided the development of additional features to enable them to manage these health-related concerns by themselves by using a medical examination, a depression screening test, alternative therapies, and more useful male partner–oriented tips and dialogues. Particularly, Dr. Joy had a male partner–friendly UI access point for use in paternal fetal education features: (1) know-how in fetal education and (2) fathers can do it (Figure 3 and Multimedia Appendix 3). Following Dr. Joy’s instruction, would-be fathers or current fathers who were inexperienced in fetal education with their partners could perform step-by-step prenatal care. To promote male partners’ involvement during routine prenatal care for a positive outcome in labor and delivery, Dr. Joy explained the need for partner support in a friendly tone and delivered practical strategies with relevant images in which a man actively supported his partner, showing empathic concerns and sympathetic responses to the men’s difficult situation related to their pregnant partners and their social life.

### Study Design

To measure perinatal women’s and their partners’ perceptions of the utilitarian and hedonic value of a medical chatbot experience, we conducted a 7-day contextual UT after completing the development of a Q&A knowledge database-based chatbot on KakaoTalk, named “Dr. Joy,” for solving their obstetric and mental health problems. This study was approved by the institutional review board of CHA Bundang Medical Center, CHA University.

#### Recruitment

In this study, two different convenience sampling methods were used to prevent this study sample from being biased and to collect samples from the population of interest. According to the result of previous research by Nielsen and his colleagues [30], 5 users has found 85% of the usability problems, and at least 15 users were needed to discover all the usability problems. As the aim of UT was to improve the chatbot design based on the usability problems, a total of 15 participants were recruited. Of 15 participants, 6 (40%) were patients who were recruited from the outpatient clinic in the Department of Obstetrics and Gynecology, CHA Bundang Women’s Hospital, CHA University. The rest (9/15, 60%) were recruited using the snowball sampling method, and therefore, 1 out of the 9 participants was asked for further potential participants who were patients at local hospitals. As Dr. Joy’s medical knowledge database could cover perinatal women’s questions ranging from antenatal care to postpartum care, women in pregnancy preparation and different pregnancy stages (ie, first [1-3 months: 1-12 weeks], second [4-7 months: 13-28 weeks], and third [8-10 months: 29-40 weeks] trimester and birth [puerperium: within 6 weeks after childbirth]) and their spouses were enrolled to complement the answers to both female and male partners’ questions in this study. Particularly, 2 married couples, who were in first and second trimester, achieved pregnancy through infertility treatments.

Following the inclusion and exclusion criteria for recruitment, the women who gave birth but were not in the 6-week puerperal period were not eligible to participate in the study. However, if the ineligible women had a plan on pregnancy immediately after puerperium, their participation was allowed as women in pregnancy preparation.

#### Usability Testing: Task and Procedure

All enrolled participants completed the 7-day long UT during the entire study period, from September 30, 2019, to October 11, 2019. All the participants were given the daily tasks of asking Dr. Joy at least 3 questions at any time and place and then giving the chatbot either positive or negative feedback with emoji (Figure 2 and Multimedia Appendix 2), using at least one feature of the obstetrics chatbot, and finally sending a facilitator all screenshots for the history of the day’s use via KakaoTalk before midnight. To make Dr. Joy available on their mobile phones, the participants were first required to search its name on the KakaoTalk Channel and add it as a friend, in order to readily access the chatbot service whenever they wanted to use it. One day after the UT completion, all participants were asked to fill out a questionnaire containing demographic characteristics, closed-ended questions about usability, perceived benefits and risks, and intention to seek and share health information on the chatbot, and open-ended questions about the strengths and weaknesses of its use.

#### Measurements

To measure the subjective usability of our newly developed chatbot service, the USE Questionnaire [28] was employed. The 30-item USE questionnaire examined the 4 subfactors of usability: usefulness (8 items), EOU (11 items), EOL (4 items), and satisfaction (7 items). All the items were anchored from 1 (strongly disagree) to 7 (strongly agree), and these 4 mean scores were averaged across all participants and sex groups to calculate a total usability score. In addition to usability, perceived benefits (2 items) and risks (2 items), and intention to seek (SEE, 6 items) and share (SHA, 4 items) health information on the chatbot using KakaoTalk were measured on a 7-point Likert scale ranging from 1 (strongly disagree) to 7 (strongly agree), and all items were adapted from Li and colleagues’ net valence model [29]. Each mean score of these factors was computed for all participants and both male and female groups. Finally, the participants responded to open-ended questions about Dr. Joy’s strengths and weaknesses, which could determine whether the chatbot led them to perceive utilitarian and hedonic value from using the chatbot.

Apart from the self-reported measures of chatbot UX, a list of users’ utterances was collected from the reports in the analysis menu of the chatbot builder and the screenshots for the history of asking Dr. Joy at least 3 questions per day during the 7-day UT period. Based on the data on the specific questions or statements that triggered fallback messages as well as the users’ positive or negative feedback on given Q&A sets extracted from the obstetric and mental health–related Q&A knowledge database, we could gain insight into the practical implications of what the questions related to real interests and concerns of male and female users were.

#### Statistical Analysis

To determine whether to use a nonparametric or parametric statistical analysis for the small-size data sets (N<50), a Shapiro-Wilk normality test was performed to check the normal distribution of the data. As the normality of EOL (W_15_=0.84; *P*=.01) and perceived risks (W_15_=0.88; *P*=.04) was violated, the Spearman correlation was chosen for the final analysis.

## Results

### Participant Characteristics

As presented in Table 1, 2 men, aged 38 and 40 years (mean 39.00 years, SD 1.41 years), and 13 women, aged 27 to 43 years (mean 34.31 years, SD 3.95 years), in pregnancy preparation or different pregnancy stages were enrolled in this study: (1) men: first trimester (1/2, 50%) and second trimester (1/2, 50%); (2) women: planned natural pregnancy (4/13, 31%), first trimester (2/13, 15%), second trimester (4/13, 31%), third trimester (1/13, 8%), and puerperium (2/13, 15%). All participants (15/15, 100%) reported KakaoTalk as the most frequently used instant messenger in everyday life.

When seeking health information on pregnancy or delivery to solve medical problems, all men referred to information sourced from books (2/2, 100%). However, women reported that they referred to multiple information sources, and the main source was acquaintances (7/13, 54%), followed by the internet (4/13, 31%), books (1/13, 8%), and health professionals (1/13, 8%). Particularly when using their personal computers or mobile phones to obtain online information on pregnancy or delivery, the 2 men employed different information search strategies: keyword search (1/2, 50%) and sentence search (1/2, 50%). A majority of women employed keyword search (11/13, 85%), and the others employed sentence search (1/13, 8%) and real-time search (1/13, 8%).

**Table 1:** Demographic information on the contextual UT participants (N=15).

ID^a^	Age (years)	Sex^b^	Pregnancy stage^c^	Pregnancy/delivery information source	Web-based information search strategy via computer or mobile phone
UTI-01	34	F	PP	Internet^d^	Keyword search^e^
UTI-02	35	F	PP	Acquaintances^f^	Keyword search
UTI-03	35	F	PP	Books^g^	Sentence search^h^
UTI-04	34	F	PP	Acquaintances	Real-time search^i^
UTI-05	31	F	FT (8 weeks)	Internet	Keyword search
UTC-06A	36	F	FT (8 weeks)	Acquaintances	Keyword search
UTC-07A	38	M	FT (8 weeks)	Books	Sentence search
UTC-08B	36	F	ST (15 weeks)	Acquaintances	Keyword search
UTC-09B	40	M	ST (15 weeks)	Books	Keyword search
UTI-10	43	F	ST (17 weeks)	Internet	Keyword search
UTI-11	33	F	ST (23 weeks)	Internet	Keyword search
UTI-12	39	F	ST (24 weeks)	Health professionals^j^	Keyword search
UTI-13	27	F	TT (32 weeks)	Acquaintances	Keyword search
UTI-14	31	F	P (3 weeks after birth)	Acquaintances	Keyword search
UTI-15	32	F	P (3 weeks after birth)	Acquaintances	Keyword search

^a^Two different ID labels were assigned to differentiate couples (UTC) from individuals (UTI) [31], and those with the same uppercase letters (A or B) are a married couple.

^b^F: female; M: male.

^c^PP: pregnancy preparation (planned natural pregnancy); FT: first trimester; ST: second trimester; TT: third trimester; P: puerperium.

^d^Internet includes portal/search engines, online communities, blogs, vlogs, etc.

^e^Keyword search with simple words, search operators, hashtags, etc.

^f^Acquaintances include friends, colleagues, online community members, experienced mothers in the same postnatal care center, etc.

^g^Books include encyclopedias of pregnancy and birth, essays and articles written by medical doctors, magazines, etc.

^h^Sentence search with a single statement/question or multiple statements/questions.

^i^Real-time search means choosing and looking for attention-capturing content published in real time on the internet.

^j^Health professionals include medical doctors, nurses, etc. An acquaintance who was a medical doctor was included in health professionals.

### Quantitative Data Analysis

The results from the USE questionnaire are shown in Table 2. Among the psychometric aspects of usability, the mean score of EOL was the highest, followed by the EOU, satisfaction, and usefulness scores in this sample. Even though the number of participants was insufficient to determine statistical significance of the difference in all 4 subfactors and total usability scores across sex, male participants showed higher mean scores than female ones. Both men and women had a tendency to rate the scores of usefulness and satisfaction lower than those of EOU and EOL; these trends were also identified within the total scores of usability and its subfactors.

Despite the higher mean score of EOL in the entire participant group, the results of the Spearman correlation indicated that there were no significant associations with usefulness, EOU, satisfaction, or total usability scores (Table 3). Unlike EOL, the total usability and other 3 subfactors had significant positive associations with each other (all ρ>0.80; *P*<.001).

**Table 2:** Descriptive statistics for sex difference in responses to USE questionnaire on the medical chatbot via KakaoTalk (N=15).

Sex	Usability subfactors^a^, mean (SD)				
	USE^b^	EOU^c^	EOL^d^	SAT^e^	Total
Men (n=2)	5.43 (1.21)	6.05 (0.96)	7.00 (0.00)	5.57 (2.02)	6.01 (2.02)
Women (n=13)	4.78 (1.12)	5.23 (0.67)	6.25 (0.71)	4.80 (1.20)	5.27 (0.82)
Total (N=15)	4.87 (1.11)	5.34 (0.73)	6.35 (0.71)	4.90 (1.26)	5.37 (0.85)

^a^Usability was measured by the average score of 4 subfactors, which is presented as the “Total” score in this table. All scales were rated from 1 (strongly disagree) to 7 (strongly agree).

^b^USE: usefulness.

^c^EOU: ease of use.

^d^EOL: ease of learning.

^e^SAT: satisfaction.

**Table 3:** Spearman rank correlation analysis of associations among individual and total usability scores from USE questionnaire on the medical chatbot via KakaoTalk (N=15).^a^

Subfactors		USE^b^	EOU^c^	EOL^d^	SAT^e^	Total
**USE**						
	Correlation coefficient (ρ)	1.00	0.82	0.26	0.98	0.97
	*P* value (2-tailed)	—^f^	<.001	.36	<.001	<.001
**EOU**						
	Correlation coefficient (ρ)	0.82	1.00	0.19	0.81	0.89
	*P* value (2-tailed)	<.001	—	.51	<.001	<.001
**EOL**						
	Correlation coefficient (ρ)	0.26	0.19	1.00	0.21	0.32
	*P* value (2-tailed)	.36	.51	—	.46	.24
**SAT**						
	Correlation coefficient (ρ)	0.98	0.81	0.21	1.00	0.95
	*P* value (2-tailed)	<.001	<.001	.46	—	<.001
**Total**						
	Correlation coefficient (ρ)	0.97	0.89	0.32	0.95	1.00
	*P* value (2-tailed)	<.001	<.001	.24	<.001	—

^a^Usability was measured by the average score of 4 subfactors, which is presented as the “Total” score in this table.

^b^USE: usefulness.

^c^EOU: ease of use.

^d^EOL: ease of learning.

^e^SAT: satisfaction.

^f^Not applicable.

Regardless of sex, the total mean score for SEE showed a similar trend to that for SHA. Compared to women, who rated the SEE score similar to the SHA score, men had a tendency to rate the mean score for SEE higher than that for SHA. Apart from the rating on SHA, the ratings on perceived benefits, SEE, and even perceived risks were higher in men than in women (Table 4).

According to the results of the Spearman correlation analysis, perceived risks exhibited no significant negative associations with perceived benefits, SEE, or SHA, whereas perceived benefits exhibited significant positive associations with both SEE and SHA. As can be seen in Table 5, perceived benefits were more strongly associated with SEE (ρ=0.94; *P*<.001) than with SHA (ρ=0.70; *P*=.004).

**Table 4:** Descriptive statistics for sex difference in responses to perceived benefits and risks and intention to seek and share health information on the medical chatbot via KakaoTalk (N=15).^a^

Sex	Factors, mean (SD)			
	PB^b^	PR^c^	SEE^d^	SHA^e^
Men (n=2)	6.25 (1.06)	3.00 (0.71)	6.17 (0.47)	5.00 (1.41)
Women (n=13)	5.19 (1.03)	2.54 (1.64)	5.01 (1.21)	4.98 (1.30)
Total (N=15)	5.33 (1.06)	2.60 (1.54)	5.17 (1.20)	4.98 (1.26)

^a^All scales were rated from 1 (strongly disagree) to 7 (strongly agree).

^b^PB: perceived benefits.

^c^PR: perceived risks.

^d^SEE: intention to seek health information.

^e^SHA: intention to seek health information.

**Table 5:** Spearman rank correlation analysis of associations among scores on perceived benefits and risks and intention to seek and share health information on the medical chatbot via KakaoTalk (N=15).

Factor		PB^a^	PR^b^	SEE^c^	SHA^d^
**PB**					
	Correlation coefficient (ρ)	1.00	−0.29	0.94	0.70
	*P* value (2-tailed)	—^e^	.30	<.001	.004
**PR**					
	Correlation coefficient (ρ)	−0.29	1.00	−0.28	−0.24
	*P* value (2-tailed)	.30	—	.32	.40
**SEE**					
	Correlation coefficient (ρ)	0.94	−0.28	1.00	0.73
	*P* value (2-tailed)	<.001	.32	—	.002
**SHA**					
	Correlation coefficient (ρ)	0.70	−0.24	0.73	1.00
	*P* value (2-tailed)	.004	.40	.002	—

^a^PB: perceived benefits.

^b^PR: perceived risks.

^c^SEE: Intention to seek health information

^d^SHA: Intention to seek health information

^e^Not applicable.

### Qualitative Data Analysis

For the qualitative data analysis, thematic analysis [32,33] was conducted on user utterance data collected via two different sources, (1) kakao i open builder and (2) usability testers, in order to complement missing data and monitor the users’ responses to a single answer or 3 Q&A sets that Dr. Joy provided. The raw data of user utterances during the 7-day UT period were extracted from the reports in the analysis menu of the chatbot builder and downloaded as separate text files, and then the files were combined into two different data sets: (1) default fallback intent (ie, users’ questions or statements that triggered error messages from Dr. Joy) and (2) predefined user intent (ie, those which triggered users’ positive or negative feedback on given Q&A sets from Dr. Joy’s knowledge database).

From the data sets, initial major themes and the chatbot’s identity, strengths, and weaknesses were produced from 316 user utterances (310 questions or statements and 6 responses to chatbot’s answers in UT) and 30 open-ended responses to the post-test questionnaire after the UT was completed (15 strengths and 15 weaknesses) by the first author. More detailed descriptions of the major themes were then generated, compared, and revised by three coders (the first author and bachelors- and masters-level research assistants) before agreement between appropriate coding categories for the 5 refined minor themes and memorable quotes was reached (Textbox 1). To ensure intercoder reliability for all 5 themes, the coded transcripts on which all coders agreed were included based on an examination of coding disagreement.

**Illustrative quotes from user utterance data by theme.**

Theme 1-1: Chatbot Identity as a Social Agent

- (1) These days, I tend to fall asleep easily at night. But...I wake up in the middle of the night, feel restless for more than two hours, and then...fall asleep again. It wasn’t like this in the early first trimester of pregnancy, but since the 15th week, sleep quality has dramatically decreased. How can I improve the quality of my sleep?
  [UTI-10]
- (2) I’m 39 and pregnant with my third child. I’m so worried that my belly at 23 weeks pregnant is much bigger than that at the same week of my previous pregnancy. I’m also worried about the deep stretch marks on my belly. Anyway...my PCP said to me...that my baby and amniotic fluid volume were normal at 23 weeks of pregnancy. Is it all right if I don’t have to worry about my belly size?
  [UTI-12]
- (3) Since I was a patient with an early cervical cancer, I have eaten turmeric powder with a teaspoon three times a day after each meal. After I found I was pregnant, I didn’t eat it for 2 months. I reached a stable period of pregnancy, so I wonder if I can eat it once a day by reducing my turmeric powder intake.
  [UTI-10]

Theme 2-1: Strengths in Chatbot’s Utilitarian and Hedonic Values

- (4) It was user-friendly to use and easy to understand how to ask questions.
  [UTC-08B]
- (5) Convenience, Speed, and Usefulness!
  [UTI-11]
- (6) A wide variety of information was provided by entering only a simple keyword.
  [UTI-13]
- (7) This chatbot was easy to access, and I could ask questions at any time.
  [UTI-12]
- (8) It was fun to see more answers to others’ frequently asked questions as well as an answer to my question.
  [UTI-4]
- (9) It was so unique and enjoyable...that I could make more than one choice from other three Q&As.
  [UTI-10]

Theme 2-2: Strengths in Chatbot’s Informational Support

- (10) For me, it was a good opportunity to know basic information more accurately.
  [UTI-5]
- (11) The strengthen was that I could look forward to more reliable responses from medical doctors, not incredible information from the Internet or online communities.
  [UTI-14]
- (12) While using this chatbot, I realized that I’ve had a lot of questions since I got pregnant and that I needed a mobile application like chatbot to solve them.
  [UTC-08B]

Theme 3: Weaknesses in Chatbot’s Content Coverage

- (13) I had to keep asking questions to get the answers that I expected.
  [UTI-05]
- (14) Blunt answers to my pointed questions...
  [UTI-02]
- (15) Sometimes...this chatbot could not recognize all abbreviations commonly used. It left a lot to be desired.
  [UTI-01]
- (16) I think its database range was too narrow. It was impossible to check the information on the government policies to boost birthrate. If it has a dictionary-style user interface where I can see each of the Q&As whenever I want, I’ll spend my spare time reading them.
  [UTI-11]
- (17) How can I have a child of the desired sex?
  [UTC-06A]
- (18) What is the chance of having a girl after a boy?
  [UTC-06A]
- (19) Can I tell the sex of my baby by my belly shape?
  [UTI-03]
- (20) What is the possibility that the baby’s sex will change after the ultrasound scan?
  [UTI-02]
- (21) Although nightmares during pregnancy are a common symptom of pregnancy, it remains a little disappointing that I have not received a professional answer to that.
  [UTI-12]

#### Theme 1-1: Chatbot Identity as a Social Agent

Although Dr. Joy was a text-based Q&A chatbot whose weakness was the lack of ability to understand what users were saying and to interact with them in a natural manner, it was found that our participants tended to consider Dr. Joy as a social actor as follows:

When asking a question, excessively detailed, personal information or their stories were included in their questions as if they talked to a close friend or acquaintance (Textbox 1, quotes 1-3).

Humanlike responses to Dr. Joy’s answers were yielded appreciating her valuable recommendations and professional medical knowledge. Our participants said the following: “Thank you.”; “Yes!”; “I got it.”; “OK, I see it.”; “Sure, I will.”; “I need to keep it properly!”

#### Theme 1-2: Chatbot Identity as a Male-Friendly Agent

Even though the facilitator gave them no specific instruction on what to ask, male participants raised questions about themselves as well as their wives, and female participants also did so about their husbands as well as themselves. They asked the following: “Can men have morning sickness?”; “Should men take folic acid?”; “Is there postpartum depression for fathers?”; “Does fathers’ medication affect pregnancy?”; “Husband is really having a hard time”; “What age is considered advanced paternal age?”

#### Theme 2-1: Strengths in Chatbot’s Utilitarian and Hedonic Values

According to the reports of all user utterance data, participants tried to view other given Q&A sets rather than press the negative feedback button or produce negative utterances on all Q&A sets. Regarding the strengths of this newly developed chatbot, a response that participants had in common was that Dr. Joy had both utilitarian and hedonic values (Textbox 1, quotes 4-9).

#### Theme 2-2: Strengths in Chatbot’s Informational Support

In addition to these strong points, some participants mentioned the benefits from health-related information sourced from Dr. Joy (Textbox 1, quotes 10-12).

#### Theme 3: Weaknesses in Chatbot’s Content Coverage

The most frequently reported weak point was that Dr. Joy failed to meet all user intents and to cover a much broader range of content domains because we focused more on helping perinatal women to prevent and solve their own mental and physical problems than on offering them answers to baby-oriented questions (Textbox 1, quotes 13-16).

In particular, routine, nonmedical questions, which were difficult for health professionals to answer, were quite often asked. For example, 2 couples at 8 and 15 weeks of pregnancy wondered about the sex of a child, so they hoped that plenty of relevant content would be supplemented in the next update. Other asked questions are listed in Textbox 1 (quotes 17-21).

## Discussion

### Principal Findings

In this study, we aimed to develop and evaluate a user-friendly Q&A chatbot with quality content and expertise for perinatal women’s and their partners’ obstetric and mental health care. This study could add to the literature by comparing the developed system or the approach of other existing chatbots with that of Dr. Joy, highlighting its technical and design contributions, and providing theoretical and empirical evidence for the perception of its UX values in the field of application addressed.

As productivity has been considered as the main motivation for chatbot use [1], this “always-on” Q&A chatbot for offering 24/7 digital support to perinatal women and their partners can be an easier and more efficient way to obtain credible information and be more intuitive to the target users than conventional means (eg, books, internet search, acquaintances, and health professionals). In line with this study, previous studies tried to expand the Q&A chatbots’ own knowledge databases to ensure content quality and improve response capability. Chung and his colleagues [19,34] applied (1) the expert-based approach to create rules for the provision of the medical information and (2) the data-based approach to provide customized information based on the already established medical knowledge database for the chatbot-based health care service, thus increasing reliability. In order to create a domain-specific or context-based chatbot to provide optimal, up-to-date answers immediately, the high-quality chatbot knowledge was extracted from social networking services such as Twitter [20], online discussion forums as web communities [35], and messengers [19]. Similar to our approach, Jeong and Seo [20] proposed a keyword matching–based answer retrieval technique based on the collection of Q&A sets from Twitter by utilizing the tweet-and-reply and the tweet-and-mention pairs and the refinement of the newly collected pairs by adding them to the existing Q&A knowledge database. As these related works focused on developing the Q&A chatbots’ answer retrieval technique to provide more accurate and flexible answers to their users, the response appropriateness of each chatbot based on quantitative data such as self-report questionnaire [20] or recall and precision measurement [18,35] was evaluated. While these proposed knowledge databases and answer retrieval techniques for Q&A chatbots were appropriate to be applied to general health care or lifecare services whose target users and content coverage were not specified, there have also been a variety of informative chatbots designed for the specific purposes of supporting pregnant women and mothers or families with young children in emergency situations [6] and providing low-cost accessible fertility and preconception health education for perinatal women [36] or breastfeeding education for community health workers and mothers in under-developed areas [37].

As entertainment and social or relational benefits have been regarded as other main motivations for chatbot use [1], the chatbot can make the process of seeking medical help enjoyable and improve the relationships between couples who need social support from their partners or care for their mental state when undergoing a stressful situation. Particularly in this study, the recommendation of evidence-based digital therapeutics, fetal education, and useful tips applicable in their daily life, as well as the establishment of a specialized medical knowledge database which fits the personal interests of women and their partners, was of great importance in developing a medical chatbot to promote their physical and mental health in the perinatal period. In the development of the first version of Dr. Joy, we focused more on enhancing and assessing the utilitarian and hedonic quality of the KakaoTalk-based Q&A chatbot as follows: (1) by building and expanding its own Q&A knowledge database with questions that were collected from peer pregnant women’s and mothers’ posts in an online community for prenatal, postnatal, and maternal care via the text-mining technique and were answered by medical specialists in the field of infertility, obstetrics and gynecology, and psychiatry; (2) by suggesting 3 optional Q&A pairs in response to the question queries of women and their partners in the perinatal period via kakao’s similarity inference engine for assessing semantic similarity between the new query and the existing Q&A sets; (3) by providing them with dialogue-based procedural recommendations and helping easily apply the knowledge to either themselves or their partners; and (4) by defining the chatbot’s identity as a medical doctor and maintaining a differentiated tone, manner, and UI when responding directly to the query and when dealing with social support– and mental health–related issues. Unlike the developed chatbots and their approaches in the aforementioned studies, this study took into account three user motivations (ie, productivity, entertainment, and social or relational benefits) and two UX values (ie, utilitarian and hedonic values) at the same level in the process of developing and assessing this medical chatbot, respectively.

The main finding of this study was that both utilitarian and hedonic value could be produced by this newly developed Q&A knowledge database–based chatbot for perinatal women’s and their partners’ obstetric and mental health care during the 7-day contextual UT period. According to the results of the USE questionnaire, it was found that Dr. Joy was very easy to learn and quick to apply, while achieving a high level of usefulness, EOU, satisfaction, and total usability was not guaranteed by its high learnability. However, given the strong associations among these 3 usability subfactors and total usability scores, it can be expected that an increase in the level of one or more usability subfactors will ensure good usability. The weak association between EOL and other subfactors also reflects that this KakaoTalk-based chatbot is a flexible system used in different contexts and for different needs of individuals [28]. As perceived usefulness, as well as perceived enjoyment, can be strongly affected by content quality as one of influential determinants of usage intention [26], Dr. Joy could provide its users with more intriguing content in its multiple Q&A responses based on the Q&A knowledge database to motivate them to acquire credible knowledge, even if the response outcomes might be a little out of line with what they expected. As reflected in the responses to the open-ended question about the strengths of Dr. Joy, participants highlighted not only the hedonic value as represented by fun, pleasure, and enjoyment, but also the utilitarian value as represented by usefulness, speed and ease to use, and convenience. In terms of its weaknesses, participants who asked questions beyond the coverage of our Q&A knowledge database pointed out that Dr. Joy with medical expertise had to suggest the right set of answers that successfully aligned with user intent, thereby enhancing its users’ trust in and their continued usage intention for the chatbot [27]. In this respect, the improvement in the quality of its Q&A set contents is of utmost importance.

Another finding was that the negative association between the perceived benefits and risks of using Dr. Joy was not significantly strong enough to influence behavioral intention in a negative direction. Furthermore, Dr. Joy led its users to perceive a low level of risks that discussing health-related information on this medical chatbot via KakaoTalk would confront them with unwanted problems or that the expected benefits of doing so would not materialize. With a low possibility of trade-off between benefit and risk, the different intentions to seek and share health information on Dr. Joy were significantly associated only with the perceived benefits, not with the perceived risks. The more its users think Dr. Joy can benefit them, the more likely they are to seek and share information from it. Compared to women, who scored SEE and SHA at similar level, the men had more intention to seek health information on medical chatbot via KakaoTalk than the women. This might be because the male partners have comparatively less opportunity to access information sources than perinatal women, who have tended to seek medical help from multiple informal and formal sources [25]. As pregnant women’s partners, our male participants, whose main source of pregnancy or delivery information was books such as encyclopedias of pregnancy and birth or essays written by medical doctors, were less likely to show the tendency to double-check information from other sources by sharing Dr. Joy’s relatively more credible information verified by health professionals. In line with the findings of our previous study [23], it can be explained that female participants, who reported relying more on multiple word-of-mouth sources of information and less on health professionals, were highly likely to share many concerns that they were reluctant to discuss with their doctors in the outpatient clinic, particularly with this KakaoTalk chatbot with a humanlike medical doctor persona.

In addition to these theoretical implications, the qualitative data suggested empirical implications for developing the next version of Dr. Joy. The main Q&A feature of this version of the informative medical chatbot was based on the response selection for a single-turn conversation, thereby intending to elicit no specific conversational responses to the given Q&A sets from the users. Nevertheless, 6 (40%) out of 15 participants showed a positive, polite attitude toward the chatbot’s answers, as if the participants had asked private questions with more personal information and responded to their doctors to show that they would follow their answers in reality (Thank you; Yes!; I got it; OK, I see it; Sure, I will; I need to keep it properly). Surprisingly, none of the participants left any negative feedback or rude, abusive utterances (eg, curses or insults) to the Q&A sets that might not meet their real intent in asking questions. This might be because the participants could not completely rule out the possibility that all their utterances would be monitored by the facilitator or researchers for the purpose of the data analysis. Despite the concern of the Hawthorne effect, these behaviors might also reflect that some users perceived Dr. Joy to be a social agent to maintain a doctor-patient–like relationship with the chatbot. As the greatest advantage of this mobile chatbot is that chatbot designers and developers can readily collect the users’ dialog inputs that were not added to the dialog blocks in advance, it can be expected to update the users’ utterance data for machine learning purposes and the chatbot’s dialog outputs and conversational UI, as well as the content values that reside in the knowledge base on a regular cycle. Particularly in terms of regular updates of the contents of Q&A sets, nonmedical but pregnancy-related subjects (eg, pronatalist policies for increasing fertility and birth rate) extracted from active users’ dialog input logs should be included to increase user retention and engagement and to decrease anxiety levels by clarifying the uncertainty of conflicting information from multiple sources, based on previous studies [22-24].

Last but not least, this study found that the male partners had needs for emotional support and information in the period of pregnancy, birth, and early fatherhood, indicating that the possibility of their needs might have been implicitly disregarded, as revealed by other studies [38-40]. Most importantly, given that pregnant women’s psychological well-being and positive pregnancy experience are closely related to better partner relationships [41,42], it is important to support male partners by adding men-oriented Q&A sets from male partners’ perspectives into this new chatbot’s knowledge database, thus helping them to understand and manage the challenges of pregnancy, birth, and the postpartum period.

### Limitations and Future Direction

As this study introduced an early-stage outcome of a government-funded research and development (R&D) project whose milestone was to investigate at least 10 perinatal women’s uptake of this initial version of Dr. Joy, the sample size of the study (N=15) was too small and its sex ratio was too unbalanced to generalize the findings to a larger population and guarantee the effectiveness of the medical Q&A chatbot, in spite of both male and female participants’ positive perceptions of the chatbot. Even though it is well-known that this sample size is enough to find out the practical implications for improving the UX of this chatbot based on its end users’ real voice and log data [30], this study has further limitations as follows:

First, the user utterance data from the small sample might be insufficient to accumulate Q&A data sets of a wide variety of pregnant women’s and their partners’ questions and concerns differently expressed with their own terms and in their own problematic situations, because Dr. Joy was designed to cover a wide range of pregnancy- and delivery-related information that was classified into 6 subjects. After the update of the Q&A sets via this usability study, the aim of this R&D project is to increase the number of active chatbot users by at least 100, collect more utterance data, and keep the Q&A knowledge base up to date. Comparison between the perception of Dr. Joy before it was initially released and that after being updated will be drawn to examine its robust uptake and the favorable perception of its utilitarian and hedonic value.

Second, Dr. Joy is geared toward encouraging perinatal women relying on multiple informal information sources to obtain evidence-based information for decision support. For this reason, we only recruited a small number of targeted participants by adopting two different convenience sampling methods to refrain from recruiting only the patients who established a good rapport with the medical doctors involved in the development of Dr. Joy, or those whose main information source was solely their doctors. However, a relatively small sample is potentially biased given the nonprobability sampling method where the sample can be taken from the units of the population that are easily accessible, thus failing to accurately reflect the responses of a large population. To deal with this potential bias of the study sample, the right probability sampling methods such as simple random sampling or clustering sampling will be used with a large sample size in future studies.

Finally, considering that a full-term pregnancy lasts 38 weeks or longer, a 7-day study period is insufficient to assess whether Dr. Joy can improve the participants’ knowledge, answer their questions effectively, or be useful for certain tasks, even if the participants provided positive usability and UX ratings in this study. To answer these research questions, which remain open for future studies, a perinatal and mental health–related variable should be directly adopted in the short-term study period, or a more longitudinal evaluation should be performed. Taken together, future studies will benefit from addressing these limitations.

### Conclusions

In sum, this study provides the potential for the uptake of this newly developed Q&A knowledge database–based KakaoTalk chatbot for perinatal women’s and their partners’ obstetric and mental health care. As Dr. Joy has quality contents, which are positively linked with both utilitarian and hedonic value, its male and female users can be encouraged to adopt and use medical chatbots in a convenient, easy-to-use, and pleasant manner. To boost their intention to continue use of Dr. Joy, its Q&A sets should be periodically updated to satisfy more user intent by monitoring both male and female user utterances.

## Appendix

Multimedia Appendix 1

Multimedia Appendix 2 Original Korean screenshots of user interface workflow for a depression screening test using the 10-item Edinburgh Postnatal Depression Scale that can be administered in the prenatal period, followed by the screening test result and therapy suggestions.

Multimedia Appendix 3 Original Korean screenshots of additional features with which male partners can provide their pregnant partners with social support that is needed for physical and mental health care, or women can take care of themselves.

## Abbreviations

AI: artificial intelligence
EOL: ease of learning
EOU: ease of use
MIM: mobile instant messenger
ob/gyn: obstetrician-gynecologist
Q&A: question-and-answer
R&D: research and development
SEE: intention to seek health information
SHA: intention to share health information
UI: user interface
USE: Usefulness, Satisfaction, and Ease of Use
UT: usability testing
UX: user experience
