# Supplementary figures and images for: A Chatbot for Perinatal Women’s and Partners’ Obstetric and Mental Health Care: Development and Usability Evaluation Study
Source: JMIR Med Inform. 2021 Mar 3;9(3):e18607. doi: 10.2196/18607 (PMC7970298; doi:10.2196/18607)

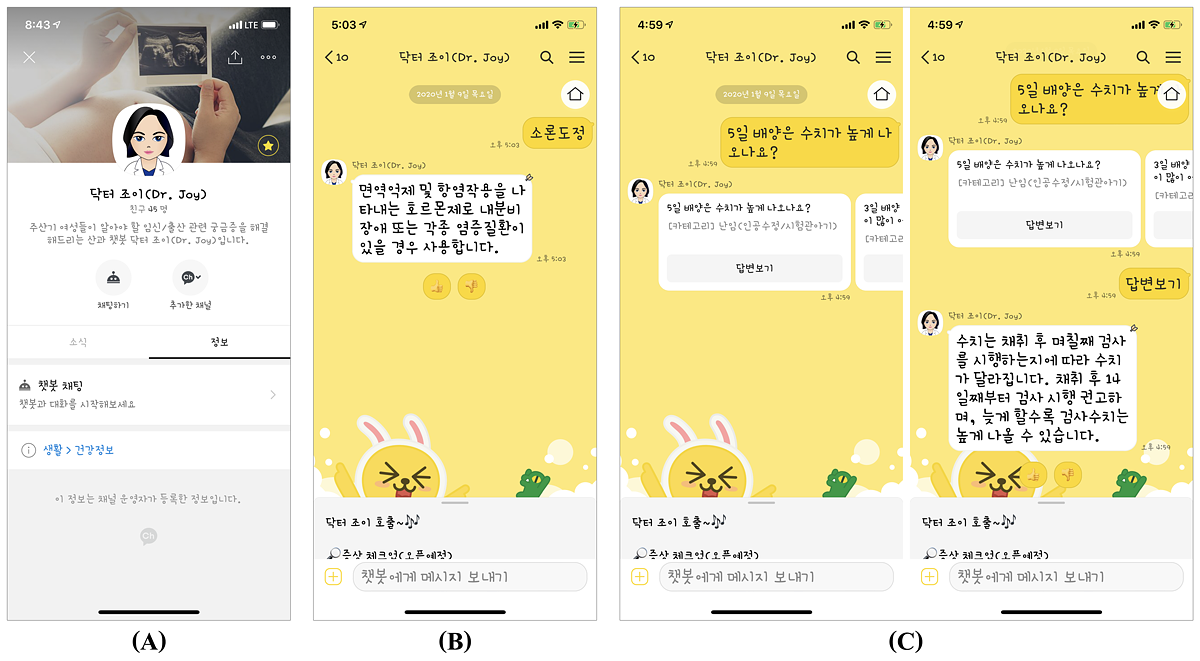

Supplement: Multimedia Appendix 1 [file medinform_v9i3e18607_app1.png]

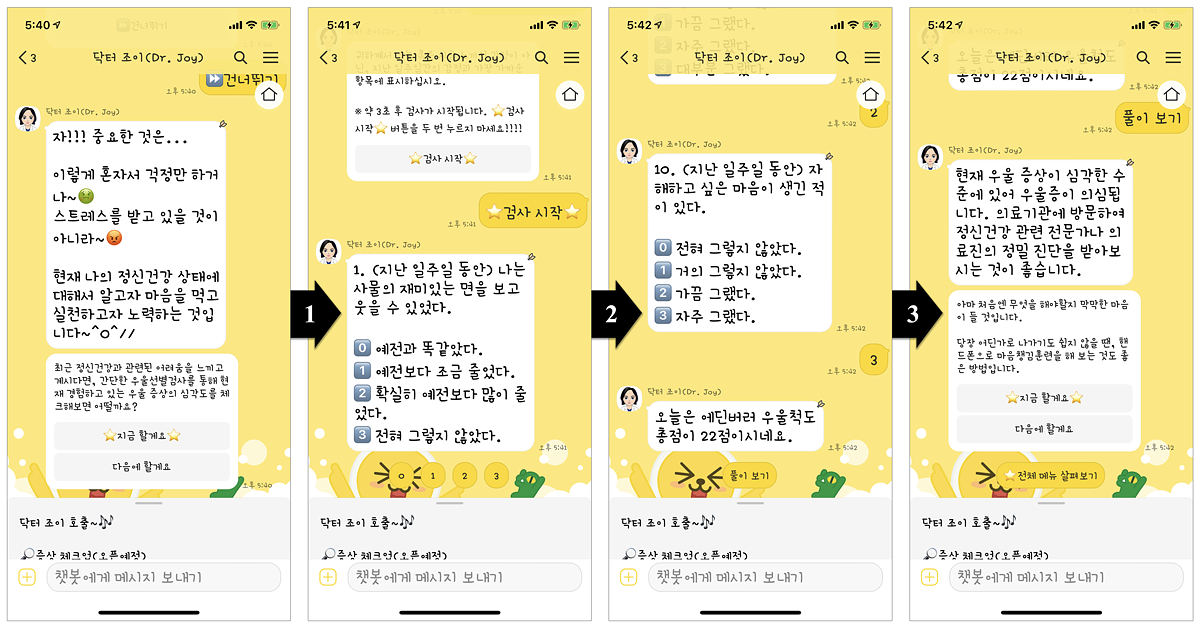

Supplement: Multimedia Appendix 2 [file medinform_v9i3e18607_app2.png]

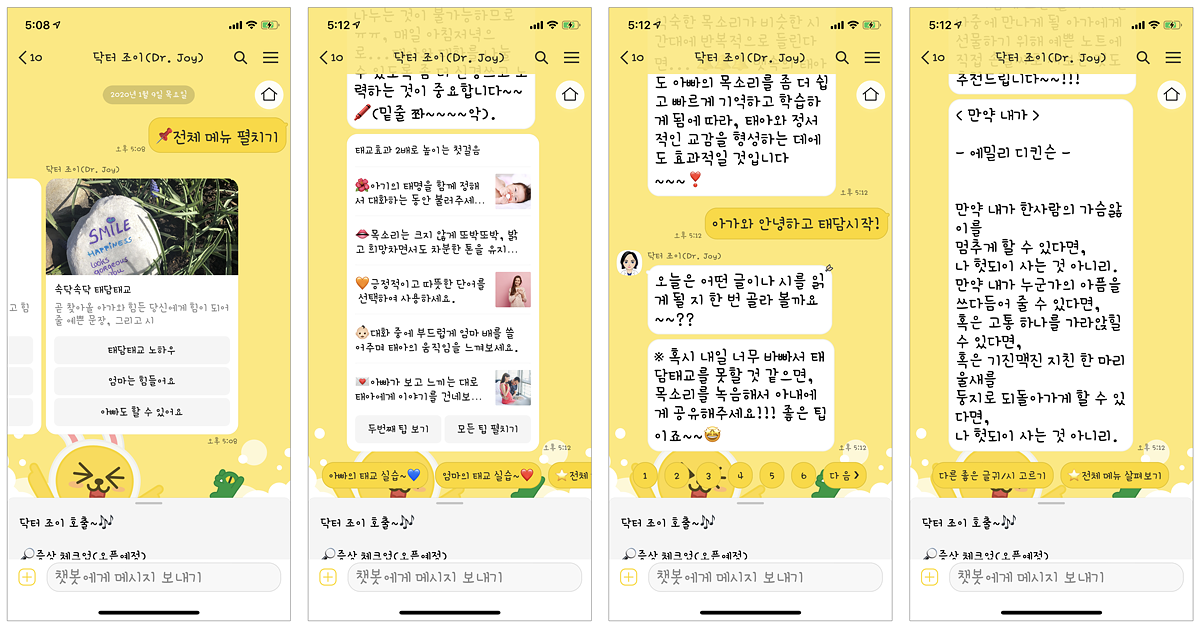

Supplement: Multimedia Appendix 3 [file medinform_v9i3e18607_app3.png]
